# Supplementary figures and images for: Functional diversity of PFKFB3 splice variants in glioblastomas
Source: PLoS One. 2021 Jul 7;16(7):e0241092. doi: 10.1371/journal.pone.0241092 (PMC8263283; doi:10.1371/journal.pone.0241092)

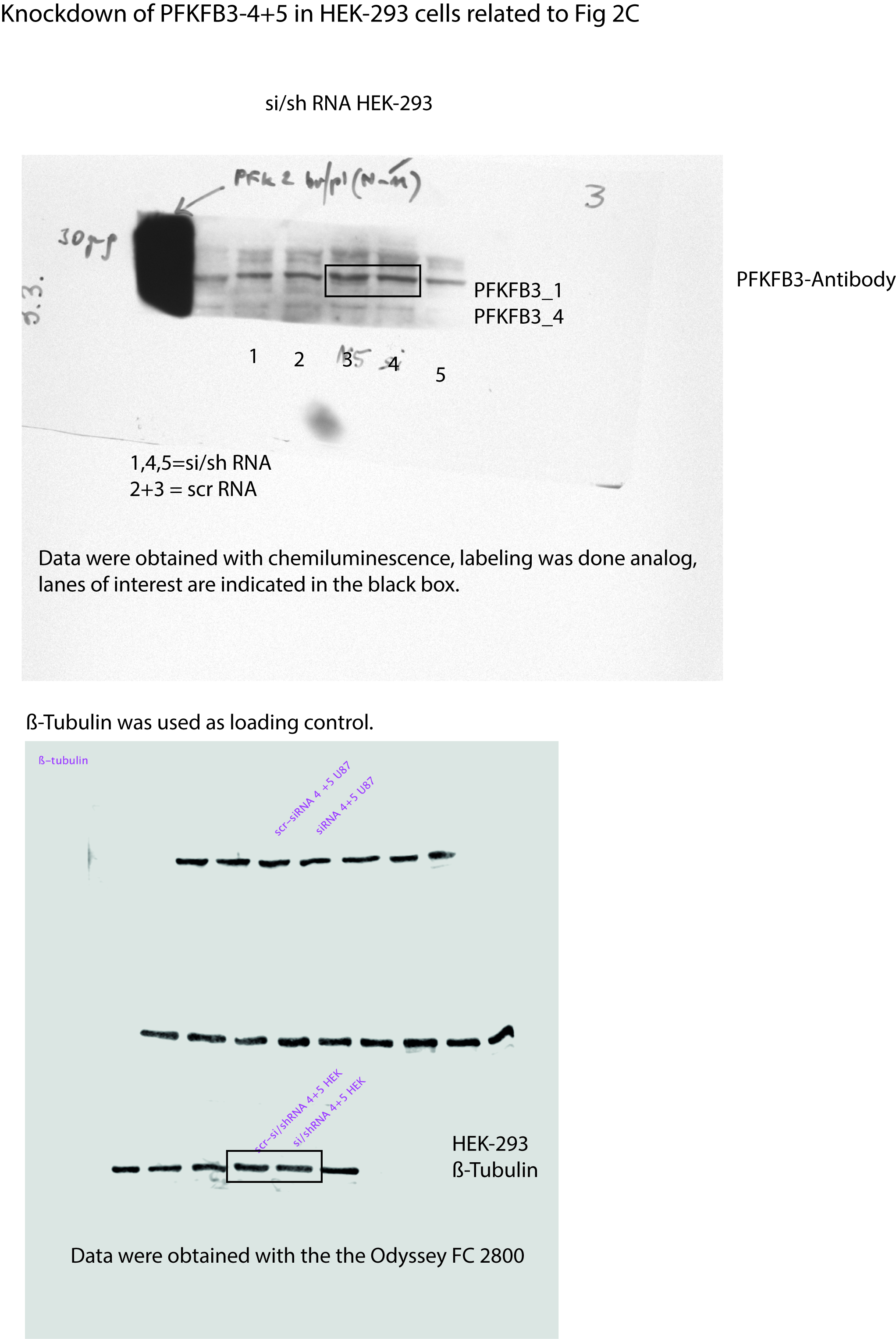

Supplement: S1 Appendix — Western blot analysis of PFKFB3 protein expression following si/shRNA mediated PFKFB3-4+5 knock-down utilizing polyclonal PFKFB3 antibody. Proteins were detected 48 h after siRNA treatment. β-Tubulin served as loading control. Raw image: S1 Appendix. (TIF) [file pone.0241092.s001.tif]

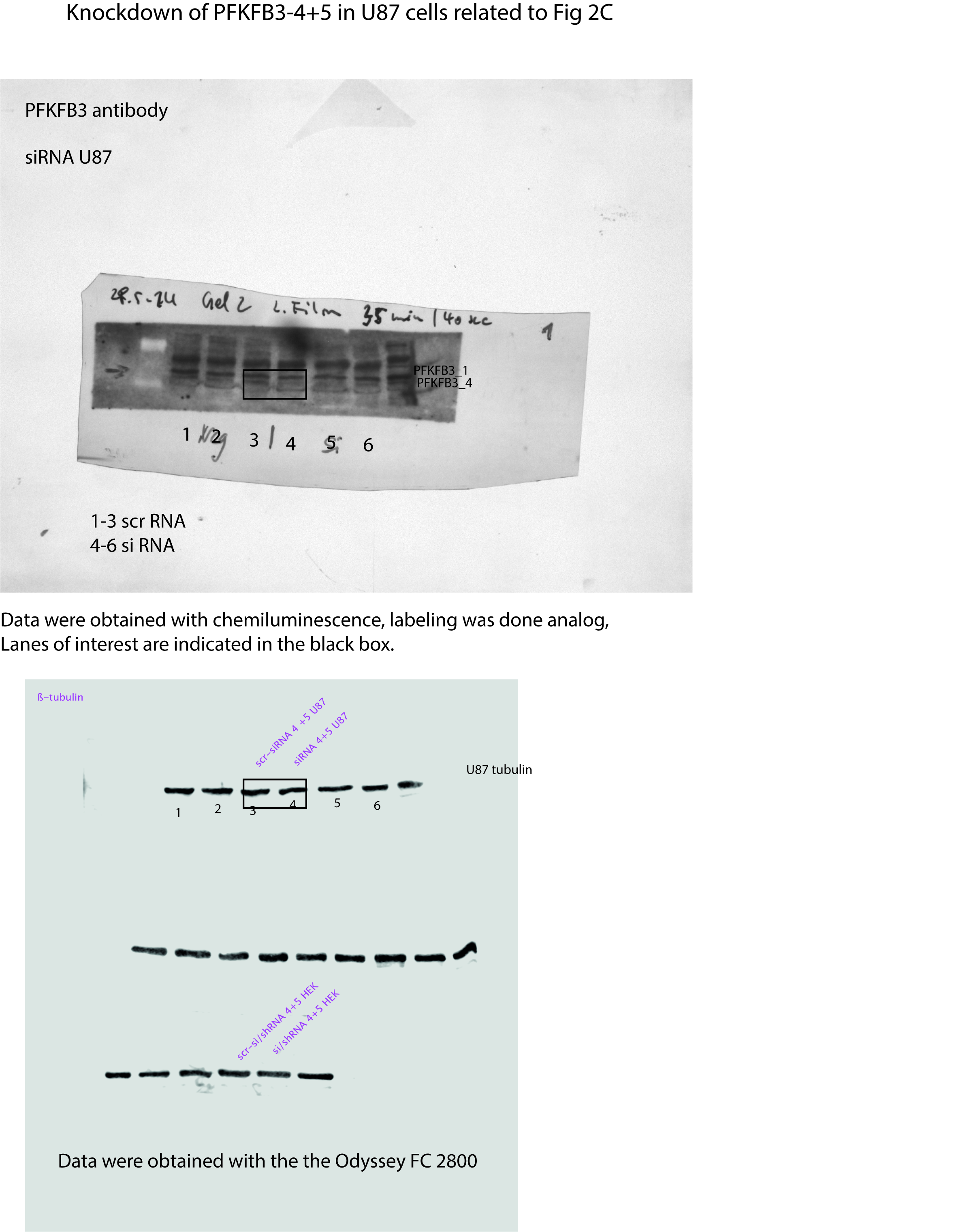

Supplement: S2 Appendix — Western blot analysis of PFKFB3 protein expression following si/shRNA mediated PFKFB3-4+5 knock-down utilizing polyclonal PFKFB3 antibody. Proteins were detected 48 h after siRNA treatment. β-Tubulin served as loading control. Raw image: S2 Appendix. (TIF) [file pone.0241092.s002.tif]

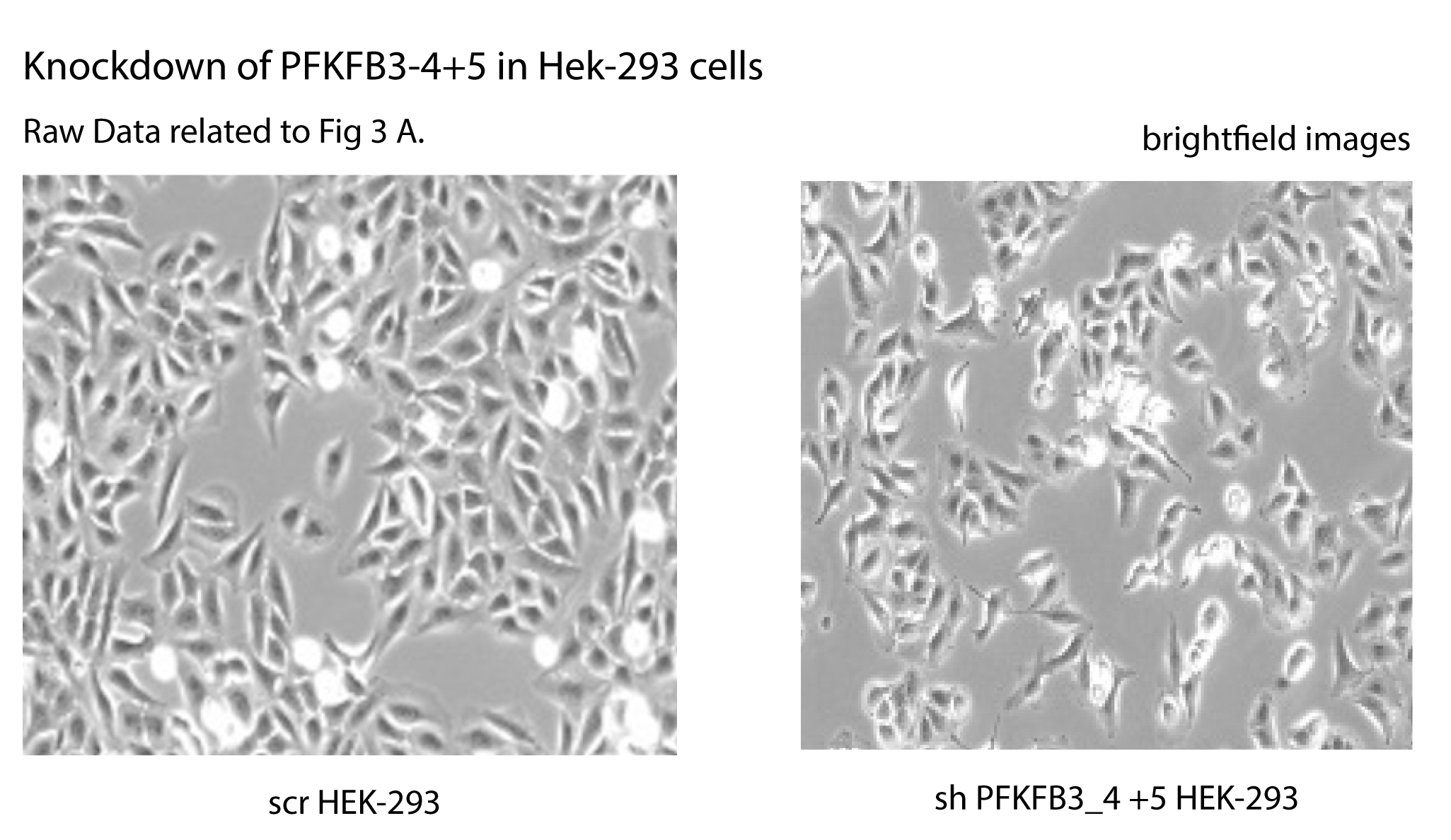

Supplement: S3 Appendix — Representative brightfield image of scr-shRNA and 4+5 shRNA treated HEK-293 cells cultured in 12-well plates. Image was taken after 5 d of cell seeding. Raw image: S3 Appendix. (TIF) [file pone.0241092.s003.tif]

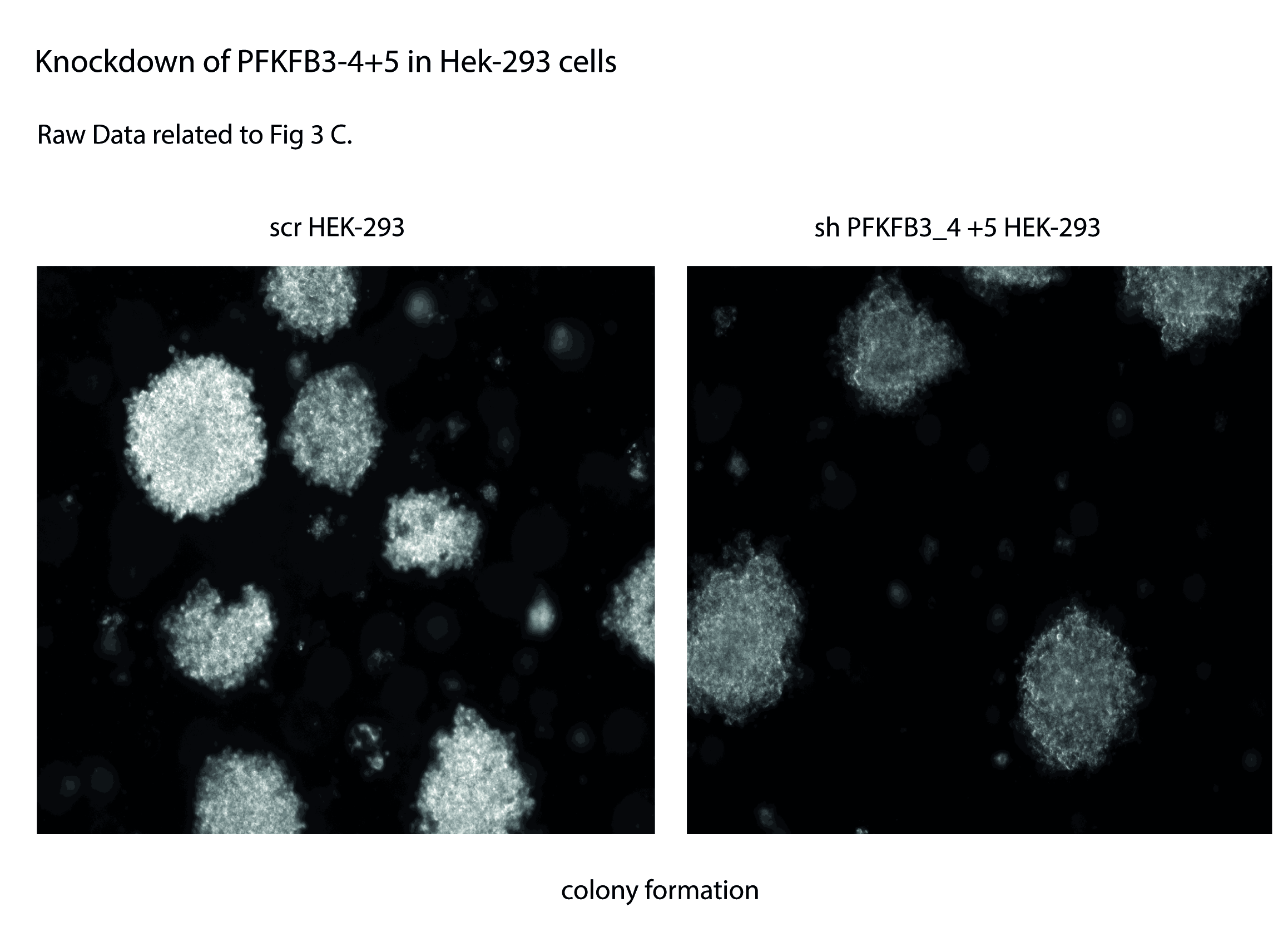

Supplement: S4 Appendix — Representative images of soft agar colonies formed by HEK-293 cells (scr-shRNA) and HEK-293 cells with stably reduced PFKFB3-4+5 levels (4/5 shRNA). The cells (5000 cells) were cultured for 14 d in 6-well plates on soft agar. Raw image: S4 Appendix. (TIF) [file pone.0241092.s004.tif]

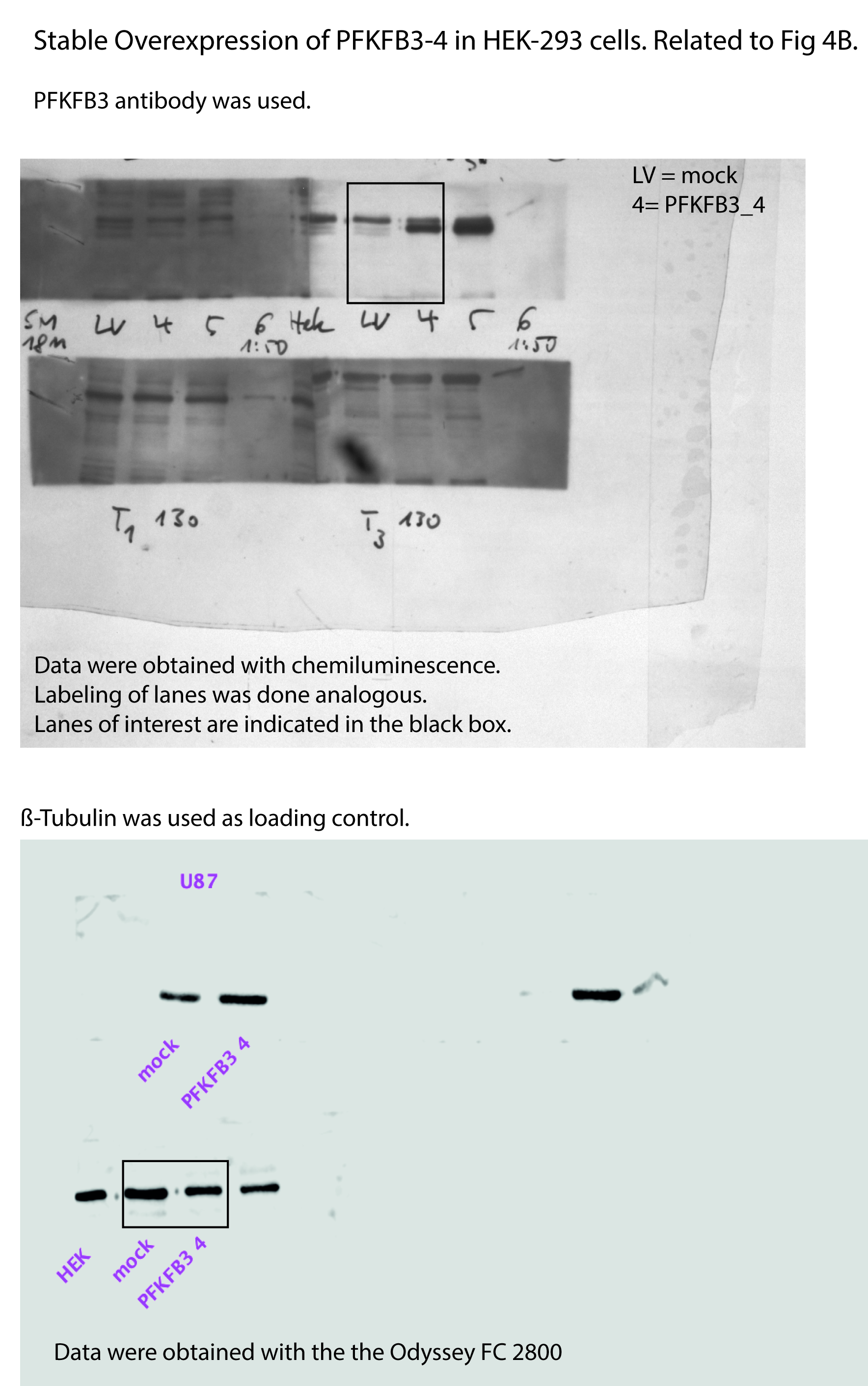

Supplement: S5 Appendix — Western blot analysis to confirm overexpression of PFKFB3-4 with polyclonal PFKFB3 antibody. β-Tubulin served as loading control. Raw image: S5 Appendix. (TIF) [file pone.0241092.s005.tif]

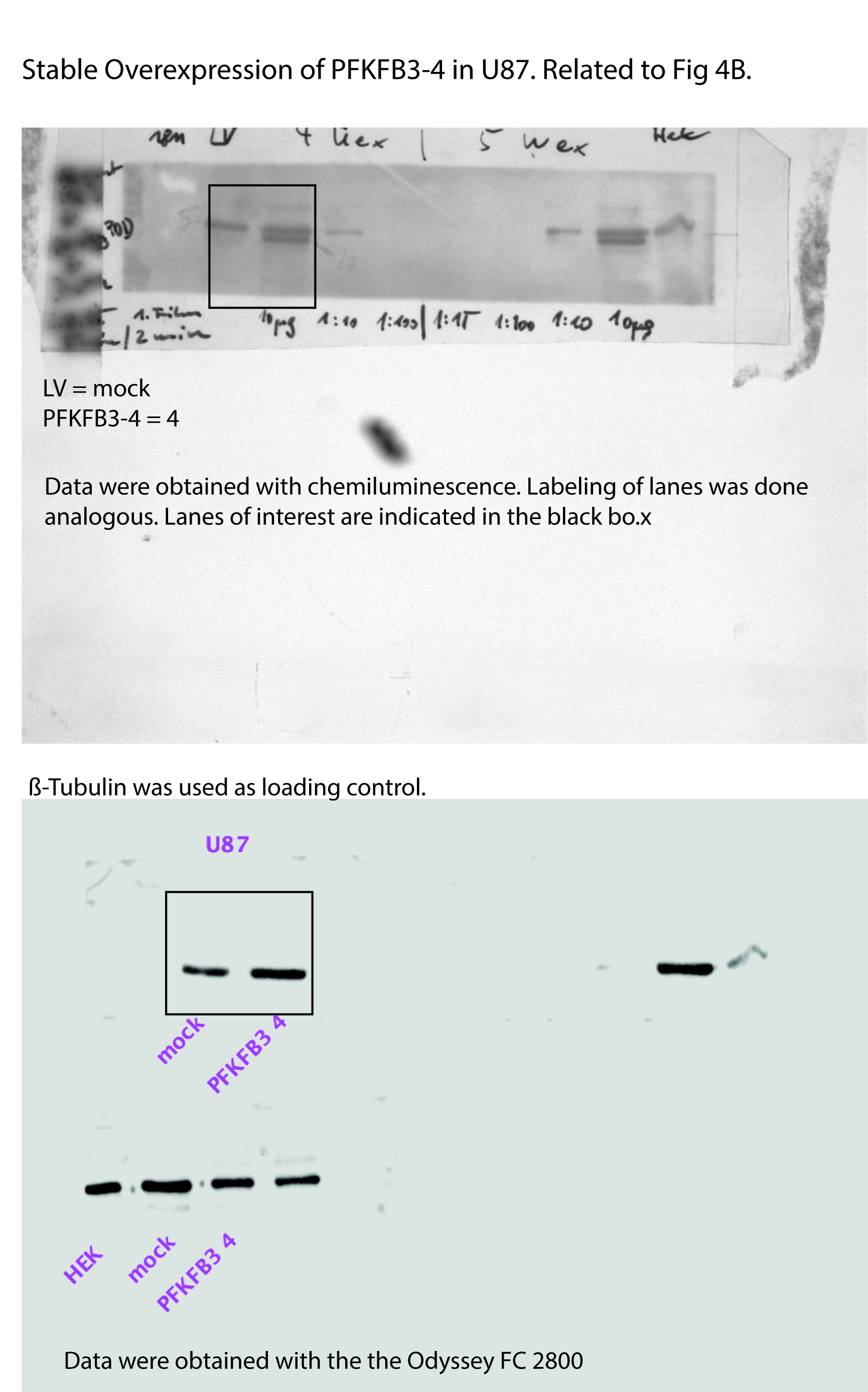

Supplement: S6 Appendix — Western blot analysis to confirm the overexpression of PFKFB3-4 with polyclonal PFKFB3 antibody. β-Tubulin served as loading control. Raw image: S6 Appendix. (TIF) [file pone.0241092.s006.tif]

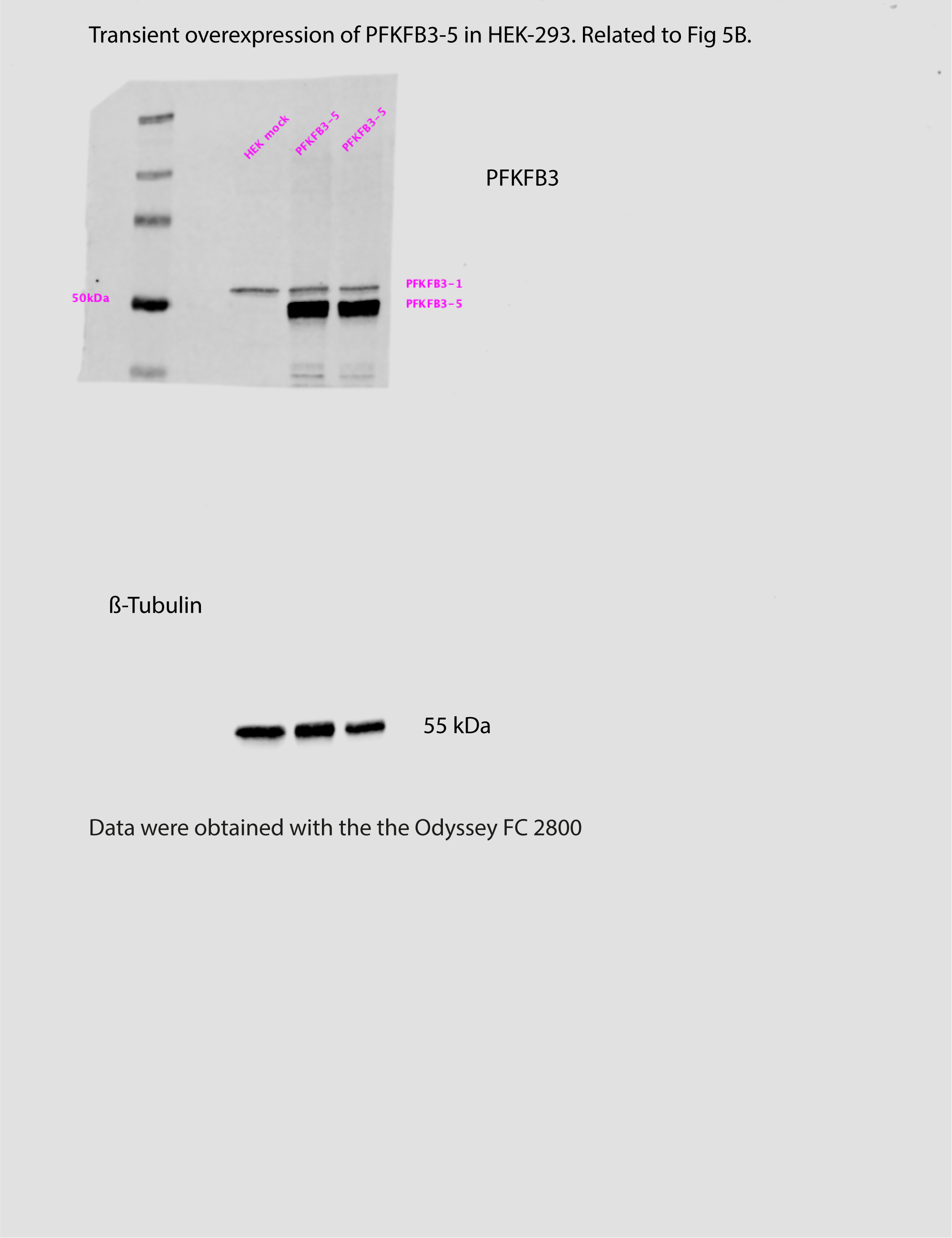

Supplement: S7 Appendix — Western blot analysis to confirm the overexpression of PFKFB3-5 with polyclonal PFKFB3 antibody. β-Tubulin served as loading control. Raw image: (S7 Appendix). (TIF) [file pone.0241092.s007.tif]

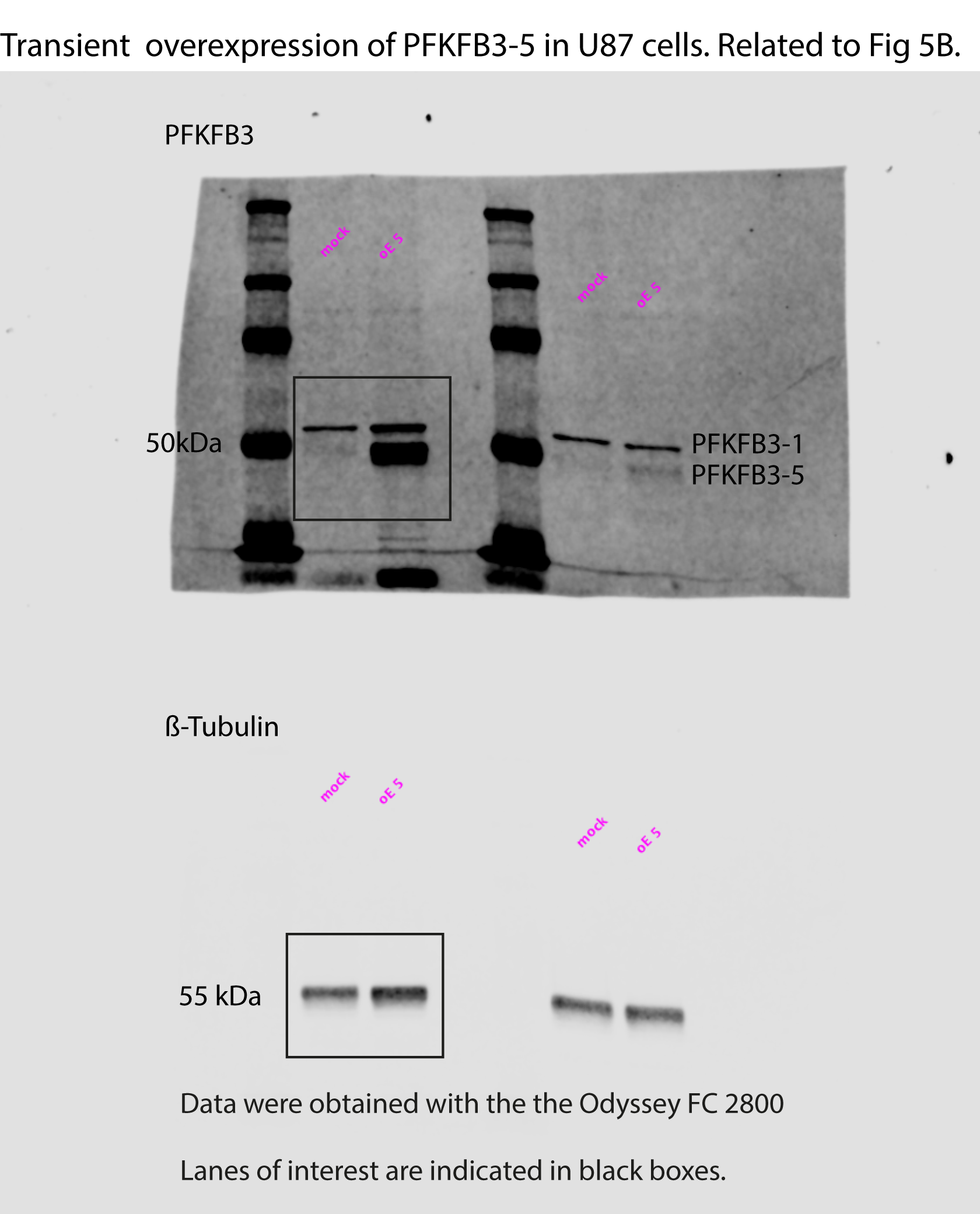

Supplement: S8 Appendix — Western blot analysis to confirm the overexpression of PFKFB3-5 with polyclonal PFKFB3 antibody. β-Tubulin served as loading control. Raw image: S8 Appendix. (TIF) [file pone.0241092.s008.tif]

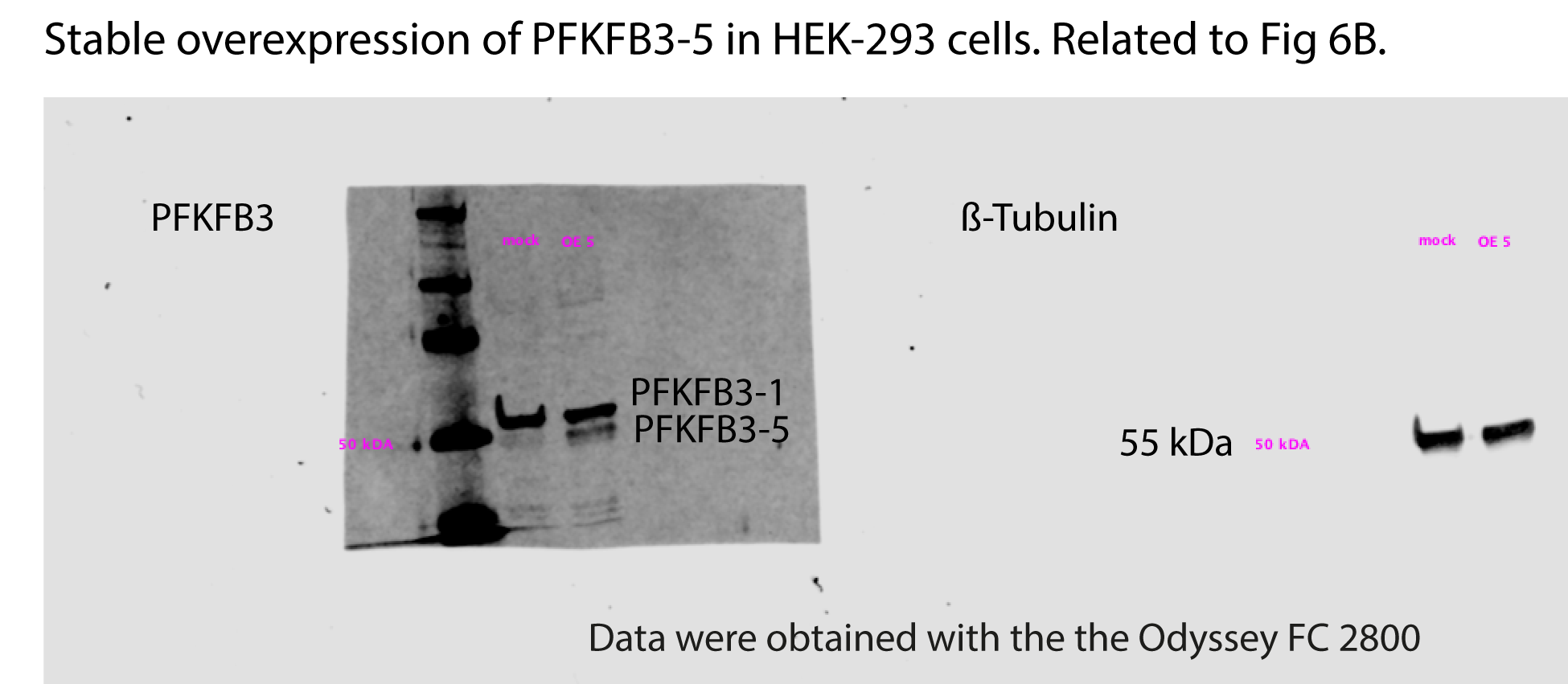

Supplement: S9 Appendix — Western blot analysis to confirm the overexpression of PFKFB3-5 with polyclonal PFKFB3 antibody. β-Tubulin served as loading control. Raw image: S9 Appendix. (TIF) [file pone.0241092.s009.tif]

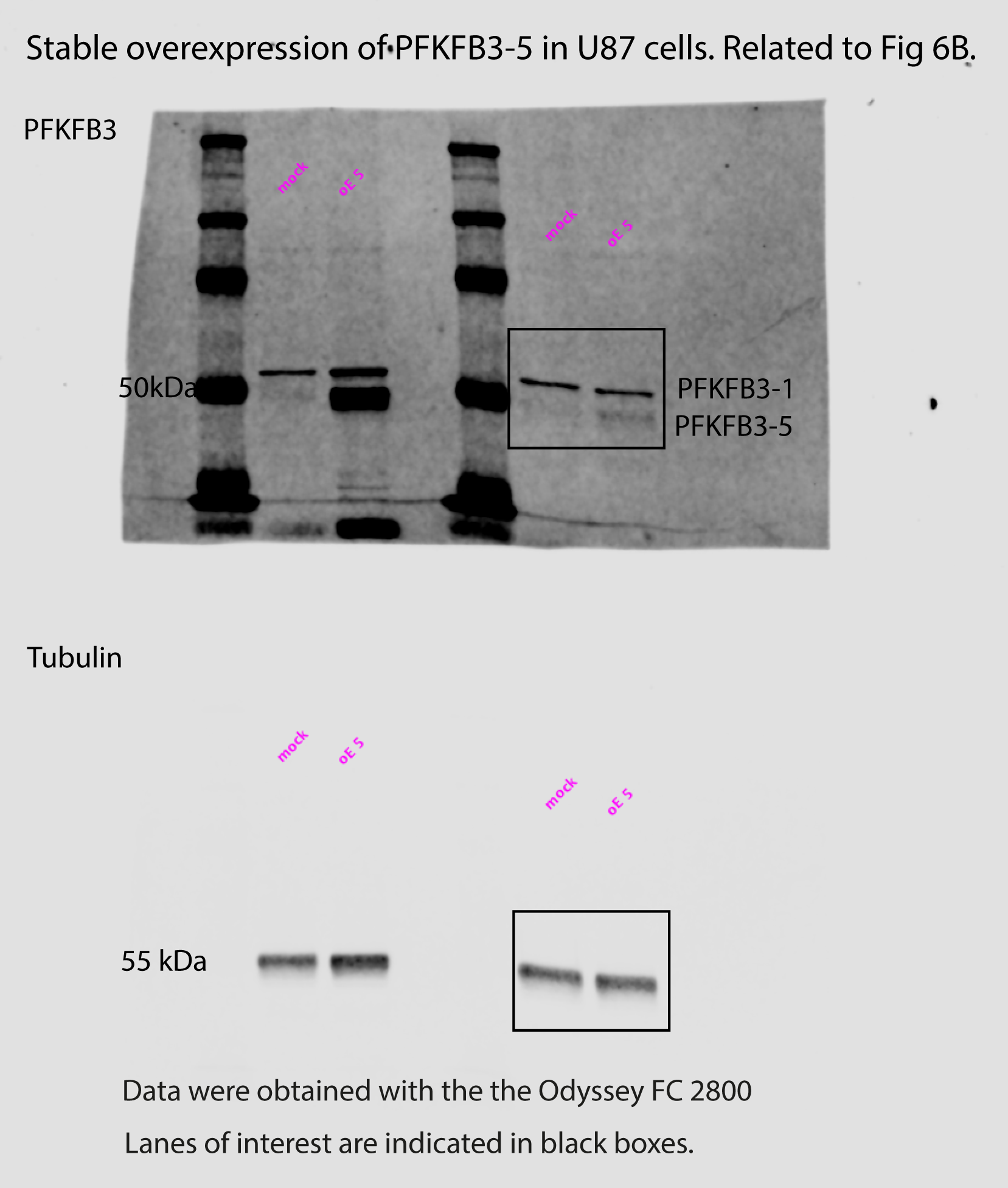

Supplement: S10 Appendix — Western blot analysis to confirm the overexpression of PFKFB3-5 with polyclonal PFKFB3 antibody. β-Tubulin served as loading control. Raw image: S10 Appendix. (TIF) [file pone.0241092.s010.tif]

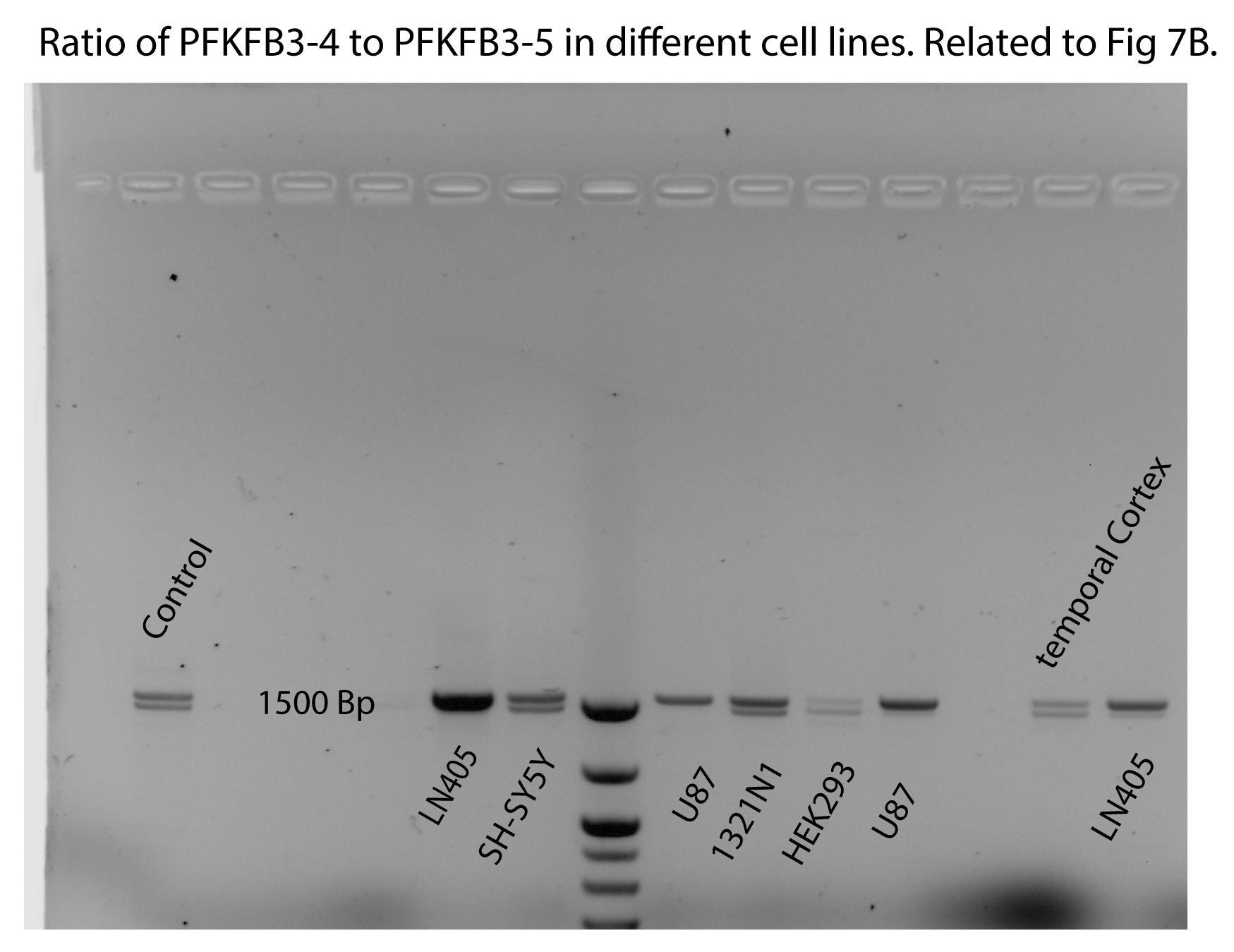

Supplement: S11 Appendix — Multiplex PCR products from several cell lines and human temporal cortex (TC) were separated by agarose gel electrophoresis. Equal amounts of mRNA of PFKFB3-4 and PFKFB3-5 (107 copies) were used as a standard (1568 bp and 1491 bp). Basis for data shown in Fig 7B and 7C. Raw image: S11 Appendix. (TIF) [file pone.0241092.s011.tif]

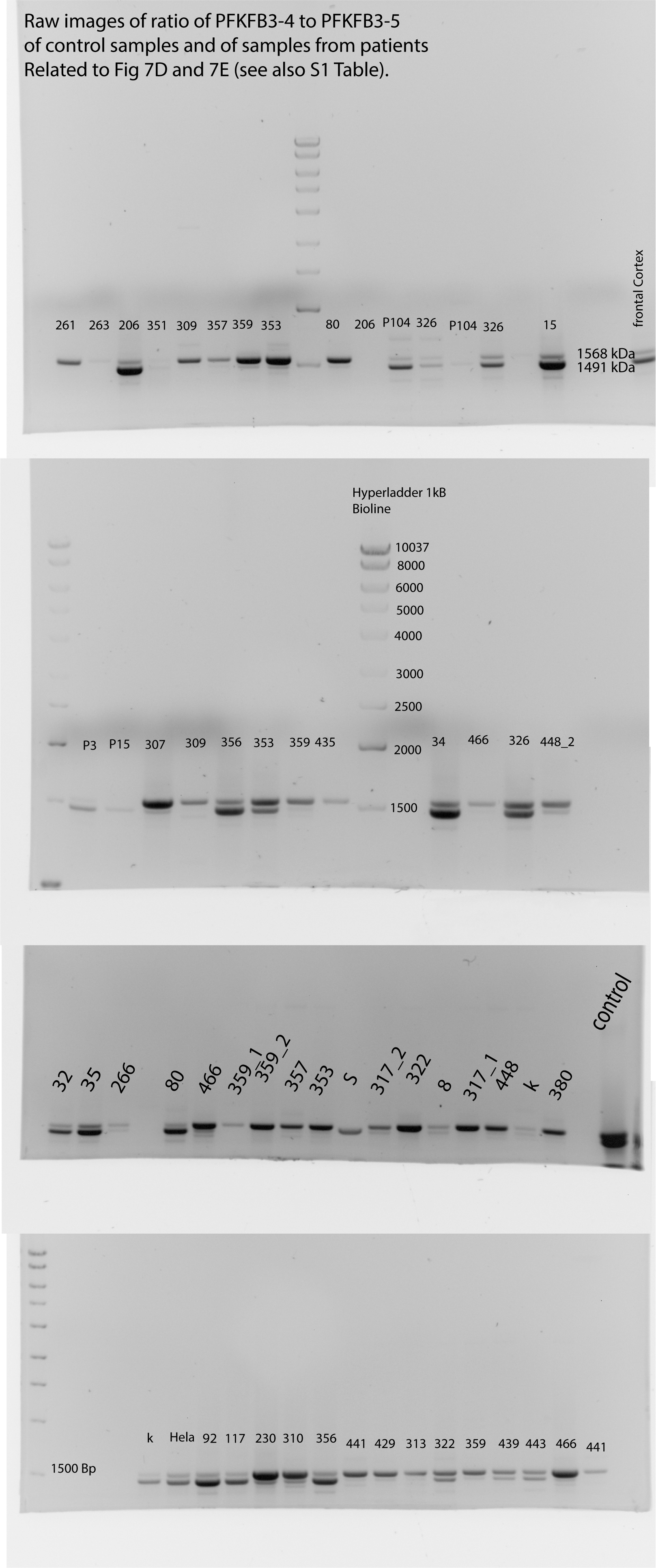

Supplement: S12 Appendix — Agarose gel electrophorese of multiplex PCR products healthy tissue as control samples and IDH-wildtype glioblastomas. Equal amounts of mRNA from PFKFB3-4 and PFKFB3-5 (107 copies) were used as a standard. Basis for data shown in Fig 7D and 7E. Raw images: S12 Appendix. (TIF) [file pone.0241092.s012.tif]
