# Supplementary material for: Functional diversity of PFKFB3 splice variants in glioblastomas
Source: PLoS One. 2021 Jul 7;16(7):e0241092. doi: 10.1371/journal.pone.0241092 (PMC8263283; doi:10.1371/journal.pone.0241092)
Supplement: S1 Table — (PDF) [file pone.0241092.s013.pdf]

S1 Table. Summraizes tumor and control samples.

Pat.-No.	WHO	Age/Gender	IDH-1	IDH-2
**5**	NN	57/f	wildtype	wildtype
**8**	NN	50/m	wildtype	wildtype
**14**	NN	30/m		
**15**	NN	30/m		
**32**	NN	27/m		
**34**	NN	46/m		
**35**	NN	40/m		
**80**	NN	73/f	wildtype	wildtype
**92**	NN	49/f		
**117**	NN	46/m		
**P3**	NN	55/m		
**P15**	NN	49/f		
**206**	NN	52/f	wildtype	wildtype
**P104**	NN	64/f	wildtype	wildtype
**326**	NN	62/f	wildtype	wildtype
**230**	IV	35/f	wildtype	wildtype
**261**	IV	66/m	wildtype	wildtype
**266**	IV	67/m	wildtype	wildtype
**307**	IV	62/f	wildtype	wildtype
**309**	IV	73/m	wildtype	wildtype
**310**	IV	56/m	wildtype	wildtype

**313**	IV	72/m	wildtype	wildtype
**317**	IV	78/f	wildtype	wildtype
**322**	IV	58/m	wildtype	wildtype
**353**	IV	57/m	wildtype	wildtype
**356**	IV	69/f	wildtype	wildtype
**357**	IV	51/f	wildtype	wildtype
**359**	IV	75/m	wildtype	wildtype
**317**	IV	78/f	wildtype	wildtype
**322**	IV	58/m	wildtype	wildtype
**353**	IV	57/m	wildtype	wildtype
**356**	IV	69/f	wildtype	wildtype
**357**	IV	51/f	wildtype	wildtype
**359**	IV	75/m	wildtype	wildtype
**359**	IV	75/m	wildtype	wildtype
**429**	IV	46/m	wildtype	wildtype
**435**	IV	45/f	wildtype	wildtype
**439**	IV	42/m	wildtype	wildtype
**441**	IV	72/f	wildtype	wildtype
**443**	IV	68/m	wildtype	wildtype
**444**	IV	62/f	wildtype	wildtype
**448**	IV	64/m	wildtype	wildtype
**466**	IV	63/f	wildtype	wildtype
**448**	IV	64/m	wildtype	wildtype
**466**	IV	63/f	wildtype	wildtype
