# Supplementary material for: Functional diversity of PFKFB3 splice variants in glioblastomas
Source: PLoS One. 2021 Jul 7;16(7):e0241092. doi: 10.1371/journal.pone.0241092 (PMC8263283; doi:10.1371/journal.pone.0241092)
Supplement: S2 Table — (PDF) [file pone.0241092.s014.pdf]

S2 Table. Lists primers and oligonucleotides used.

Name	Sequence (5'-3')
Genotyping	
IDH1f	CGGTCTTCAGAGAAGCCATT
IDH1r	GCAAAATCACATTATTGCCAAC
IDH2f	CCAATGGAACTATCCGGAAC
IDH2r	TGTGGCCTTGTACTGCAGAG
Plasmid construction shRNA	
shPFKFB3-4/5 sense	GATCCCGCCTGTCTAACATGAAAGGTTCAAGAGACCTTTCATGTTAGACAGGCTTTTTGGAAA
shPFKFB3-4/5 antisense	AGCTTTTCCAAAAAGCCTGTCTAACATGAAAGGTCTCTTGAACCTTTCATGTTAGACAGGCGG
scr sense	GATCCCAGTACTGCTTACGATACGGTTCAAGAGACCGTATCGTAAGCAGTACTTTTTTGGAAA
scr antisense	AGCTTTTCCAAAAAAGTACTGCTTACGATACGGTCTCTTGAACCGTATCGTAAGCAGTACTGG
Plasmid construction overexpression	
PFKFB3-4 f	TGGGCCCAAGATGCCGTTGGAACTGACGCAGAG
PFKFB3-4 r	ATACTTAAGTCATGTTAGACAGGCTTGCC
PFKFB3-5 f	ACTTAAGCTTATGCCCTTCAGGAAA
PFKFB3-5 r	TAGCTCTAGATCATGTTAGACAGGCTTG
qPCR	
3PFK2fo2	GAGACGCAATAGTGTCAC
iPFK2re6	GGAGCCTTTCATGTTAGAC

TBPfo	TTCCACTCACAGACTCTCAC
TBPre	GCTCTCTTATCCTCATGATTACC
iPFK2fo	ACACCGGGAGAGGTCAGAGGATG
iPFK2re	CTACGGGAGCCTTTCATGTTTTG
HB10	GAATGTGCTGGTCATCTGCC
6PFK32re5	GAGCCTTTCATGTTCTCTG
Multiplex PCR	
4_Fo	ATGCCGTTGGAACTGACGCAG
5_Fo	GCGCGATGCCCTTCAGGAAAG
4+5-Re	ACAGGCTTGCCCTAGCAAAGG
