# Supplementary material for: Functional diversity of PFKFB3 splice variants in glioblastomas
Source: PLoS One. 2021 Jul 7;16(7):e0241092. doi: 10.1371/journal.pone.0241092 (PMC8263283; doi:10.1371/journal.pone.0241092)
Supplement: S3 Table — (PDF) [file pone.0241092.s015.pdf]

S3 Table. This table shows siRNAs used.

Name	Sequence (5’-3`)
scr-siRNA sense	UGGUUUACAUGUCGACUAA
scr-siRNA antisense	UUAGUCGACAUGUAAACCA
PFKFB3-4/5 siRNA-sense	GGGCAAGCCUGUCUAACAU
PFKFB3-4/5 siRNA-antisense	AUGUUAGACAGGCUUGCCC
